# Functional Characterization of Different *Fructilactobacillus sanfranciscensis* Strains Isolated from Chinese Traditional Sourdoughs

**DOI:** 10.3390/foods13172670

**Published:** 2024-08-24

**Authors:** Huanyi Yang, Jiaqi Lin, Xueyuan Han, Juguo Bi, Lijia Dong, Jianqiu Sun, Chi Shen, Ying Xu

**Affiliations:** 1 School of Life and Environmental Science, Shaoxing University, Shaoxing 312000, China; yanghysxu@126.com (H.Y.); ljq2733744166@163.com (J.L.); 18867146394@163.com (X.H.); 19518557610@163.com (J.B.); donglijia@126.com (L.D.); jianqius@163.com (J.S.); sc@usx.edu.cn (C.S.); 2 College of Food Science and Engineering, Ocean University of China, Qingdao 266003, China

**Keywords:** *Fructilactobacillus sanfranciscensis*, phenotypic diversity, proteolysis, acidifying activity, volatile compound, transcriptome

## Abstract

*Fructilactobacillus sanfranciscensis*, the dominant species of lactic acid bacteria in sourdoughs, impacts the microstructure and flavor of steamed bread through exopolysaccharide production, acidification, proteolysis, and volatile compound generation. The aim of this study is to investigate the phenotypic diversity and technological traits of 28 *F. sanfranciscensis* strains of different genotypes isolated from Chinese traditional sourdoughs. The results showed that *F. sanfranciscensis* strains exhibited substantial variation in proteinase and peptidase activities and the amount of acidification and volatiles in fermented sourdoughs. However, we observed no significant differences in exopolysaccharide production among the strains. The strains Sx14 and Ts1 were further chosen for transcriptomics to gain a deep insight into their intraspecies diversity in sourdough fermentation. Significant transcriptome differentiations between these two strains after 12 h fermentation in sourdoughs were revealed. According to the results, the strain Sx14 possessed higher dipeptidase and aminopeptidase activities, galactose utilization, and lactic and acetic acid production abilities, whereas Ts1 showed higher transmembrane transport of substrates and fructose utilization.

## 1. Introduction

Sourdough, one of the oldest natural starter cultures, is mostly used to produce cereal-based fermented products [1,2,3]. In China, sourdough has been utilized for centuries as a leavening agent for steamed bread preparation [4,5]. Although instant yeast offers advantages for dough leavening, sourdough is preferred because of its particular taste. Sourdough fermentation contributes significantly to the overall bread quality, resulting in better aroma, flavor, and texture, as well as increased nutritional value and longer shelf life [6,7], primarily via the inherent microbes’ metabolic activities. Different investigations on sourdough microbiota have indicated that lactic acid bacteria (LAB) and yeasts dominate this niche [8,9,10,11]. The metabolic activities of LAB contribute to sourdough fermentation, mainly through acidification, flavor formation, and the production of antimicrobial agents [8].

*Fructilactobacillus sanfranciscensis* (formerly *Lactobacillus sanfranciscensis*) is an essential autochthonous species in traditional (type I) sourdough [8,12,13] that dominates over heterofermentative and homofermentative lactobacilli and is the main driver of fermentation [14]. *F. sanfranciscensis* is also the dominant LAB species in Chinese traditional sourdoughs [15,16]. Numerous studies have characterized *F. sanfranciscensis* and revealed its association with the fermentation of sourdough [13]. Exopolysaccharide (EPS) production, acidification, and proteolysis by *F. sanfranciscensis* have a substantial impact on the microstructure of sourdough and bread [2]. Moreover, *F. sanfranciscensis* enhances bread flavor by generating volatile compounds including acetic acid, aldehydes, alcohols, and ethyl acetate [17,18]. Molecular techniques, including multilocus sequence typing (MLST), pulsed-field gel electrophoresis (PFGE), random amplified polymorphic DNA (RAPD), and PCR amplification of repetitive bacterial DNA elements (rep-PCR), have been employed to indicate *F. sanfranciscensis* intraspecies diversity [14,16,19,20,21]. Understanding the metabolism of various strains of *F. sanfranciscensis* and their effect on sourdough and bread is vital for categorizing the candidate microorganisms, used to develop starter preparations with stable microbial consortia. However, after describing the genetic diversity, most studies do not provide further data on the phenotypic diversity and technological relevance of *F. sanfranciscensis* strains [19,21,22].

Therefore, in this study, we describe the phenotypic diversity and technological functions of *F. sanfranciscensis* strains obtained in our previous studies. These strains were isolated from Chinese traditional sourdoughs in different regions and exhibited genotypic variations. However, it seems that their genotypes have no correlation with geographical origin. Proteinase and peptidase contents, EPS production, acidification, and volatile matter formation during sourdough fermentation are analyzed to characterize the strains. Transcriptomics is then employed to perform gene expression analysis of different *F. sanfranciscensis* strains and elucidate their intraspecies diversity during the fermentation of sourdough.

## 2. Materials and Methods

### 2.1. Strains

In this research, we selected 28 *F. sanfranciscensis* strains based on the genotypic differentiation by multiplex-RAPD and MLST analyses performed in our previous research [16] (Table 1). All strains were kept at −80 °C in SDB broth [23] augmented with glycerol (25%; *v*/*v*).

### 2.2. Proteinase and Peptidase Activities

The proteinase and peptidase activities of the *F. sanfranciscensis* strains were determined per the protocols provided by De Angelis et al. [14], with certain modifications. All the strains were activated twice under anaerobiosis (YQX-II, Shanghai CIMO Medical Instrument Co. Ltd., Shanghai, China) for 24 h at 30 °C. For collection, the cells were centrifuged at 12,400× *g* at 4 °C for 10 min and rinsed 2× using sterile potassium phosphate buffer (0.05 M, pH 7.0). The rinsed pellet was redissolved in the same buffer at a concentration of approximately log 9 CFU mL^−1^ for enzyme analysis.

Wheat albumins and globulins, which were isolated from wheat flour per the protocol reported by Weiss et al. [24], were used as substrates to determine proteinase activity. Wheat flour (1 g) was mixed with 4 mL Tris-HCl buffer (50 mM; pH 8.8) and extracted at 4 °C for 1 h with a vortex at 15 min intervals. The supernatant containing albumin and globulin proteins was acquired by 20 min of centrifugation at 4 °C and 20,000× *g*. For determination, substrate (0.9 mL; 4 mg/mL of albumins/globulins proteins) and 0.1 mL of cellular suspension were mixed and incubated under stirring conditions (150 rpm) at 30 °C for 48 h. Subsequently, SDS-PAGE was conducted per the protocol of Gerez et al. [25] on 12% (*w*/*v*) acrylamide gels stained with Coomassie Brilliant Blue R 250 (Sigma-Aldrich, St. Louis, MO, USA).

Aminopeptidase activity was determined using Leu-*p*-nitroanilides (Sigma-Aldrich) as synthetic substrates. The analysis mixture contained cell suspension (0.2 mL) and substrate (0.9 mL; 2 mM) in potassium phosphate buffer (pH 7.0; 0.05 M). After 1 h of incubation at 30 °C, 0.6 mL of acetic acid (10%, *v*/*v*) was added to terminate the reaction, and then the absorbance was assessed at 410 nm. For data comparison, a standard curve was assessed using *p*-nitroaniline (*p*-NA). One unit (U) of activity was described as the amount of enzyme needed to release 1 µmol of *p*-NA/min under the assay conditions.

The activities of dipeptidase and tripeptidase were measured using Leu-Leu and Leu-Leu-Leu substrates, respectively (Sigma-Aldrich). The activities of dipeptides and tripeptides were assayed using the Cd-ninhydrin protocol [26]. The analysis mixture comprising cell suspension (0.2 mL) and substrate (0.5 mL; 4 mM) in potassium phosphate buffer (pH 7.0; 0.05 M) was incubated for 1 h at 30 °C. For reaction termination, 1 mL of the Cd-ninhydrin reagent was added. After 5 min of incubation at 85 °C, the solution was cooled and the absorbance was measured at 505 nm. One unit (U) of activity was described as the amount of enzyme needed to release 1 µmol of amino acid per minute. For data comparison, a standard curve was generated using leucine.

### 2.3. EPS Determination

The EPS concentration produced by the *F. sanfranciscensis* strains was determined by following the protocol reported by Liu et al. [27], with certain modifications. All strains were activated twice in SDB broth. Then, 1% of each strain (*v*/*v*) was incubated anaerobically with SDB broth (50 mL) at 30 °C for 48 h followed by 30 min centrifugation (6000× *g*, at 4 °C). The buoyant was collected, boiled for 10 min in a water bath, and then cooled in ambient temperature. Subsequently, to the above solution, trichloroacetic acid was added to make a final 10% concentration (*w*/*v*) and kept at 4 °C overnight. After 15 min centrifugation (12,000× *g* at 4 °C), the supernatant fluid was collected and three volumes of cold ethanol were added. After overnight incubation at 4 °C, the precipitate was collected by centrifugation, as above. The pellet was dissolved in deionized water and dialyzed (cut-off molecular weight: 12,000–14,000 Da) against deionized water for 72 h at 4 °C. A crude polysaccharide solution was obtained, and the neutral carbohydrate concentration was assessed via the phenol–sulfuric acid assay, keeping glucose as a standard.

### 2.4. Sourdough Fermentation

This research used wheat flour, purchased from Weifang Fengzheng Flour Co., Ltd. (Weifang, China), composed (*m/m*) of approximately 1% fat, 14% moisture, 11% protein, 72.1% carbohydrate, and 0.6% ash.

All strains were anaerobically incubated at 30 °C for 48 h in SDB broth. For acquisition, the cells were centrifuged for 15 min (at 4 °C and 5000× *g*) and rinsed twice with sterile distilled water. Distilled water, wheat flour, and the *F. sanfranciscensis* cells (inoculum size 10^7^ CFU g^−1^ dough) were thoroughly mixed in a mixer (HMJ-D3826, Guangdong Bear Electric Co., Ltd., Foshan, China) for 10 min to obtain dough with a dough yield (DY, 100× dough weight/flour weight) of 150. Then, each dough was individually inoculated with a different *F. sanfranciscensis* strain in triplicate and placed in an incubator (STIK(Shanghai) Co., Ltd., Shanghai, China) at 30 °C and relative humidity of 80% for 12 h.

### 2.5. pH, Total Titratable Acidity, and Acid Content

For each sourdough sample, the pH and total titratable acidity (TTA) were assessed, as previously described [16]. Lactic and acetic acid concentrations were measured using a D-/L-Lactic Acid Assay Kit and Acetic Acid Assay Kit (Megazyme International, Wicklow, Ireland), respectively, per the kit’s guide. The fermentation quotient (FQ) was elucidated as lactic acid-to-acetic acid molar ratio, as reported by De Angelis et al. [14].

### 2.6. Assessment of Sourdough’s Volatile Compounds

Volatile compounds in sourdoughs fermented by different *F. sanfranciscensis* strains were determined, per the method reported by Yang et al. [28]. Headspace solid-phase microextraction (HS-SPME) was utilized to extract the volatile compounds, and for separation and identification, gas chromatography–mass spectrometry (GC-MS) was employed. Samples were prepared by the addition of 3.0 g of dough and 1.0 g of NaCl to headspace vials. The Carboxen/Polydimethylsiloxane fiber assembly (diameter, 75 µm; Supelco, Inc., Bellefonte, PA, USA) was utilized for 30 min for extracting volatile compound at 60 °C and then directly administered in the injection port of the gas chromatograph (7890B; Agilent Technologies, Santa Clara, CA, USA), attached with a DB-WAX capillary column (J&W Scientific, 0.25 µm film thickness, 30 m long × 0.25 mm internal diameter), to desorb the acquired volatiles in splitless mode for 4 min at 250 °C. The column temperature was set to 40 °C for 2 min, increased to 230 °C at a rate of 5 °C/min, and was then sustained for 7 min. Helium was used as a carrier gas at a flow rate of 1.0 mL/min. The gas chromatograph was coupled to a mass spectrometry detector (5977A; Agilent Technologies) in scan mode (35–500 *m*/*z*) with an electronic impact of 70 eV. For the identification of volatile compounds, the obtained mass spectral data were compared with those from a commercial database (NIST 17).

### 2.7. mRNA Sequencing

Whole RNA was acquired from *F. sanfranciscensis* inoculated sourdough samples after 12 h of Trizol Reagent (Invitrogen, Carlsbad, CA, USA) treatment and purified using the RNeasy^®^ Mini kit (Qiagen, Hilden, Germany), per the kit’s guide. The integrity and quantity of the isolated RNA were elucidated via a Bioanalyzer 2100 system (Agilent, USA). Ribo-Zero rRNA Removal Kit (Illumina, San Diego, CA, USA) was employed to remove ribosomal RNA from total RNA. Using the Illumina TruSeq^®^ Stranded mRNA Library Prep Kit, the sequencing libraries were generated, per the kit’s protocol. For RNA-seq analysis, the Illumina HiSeq platform was used to perform paired-end sequencing and generate 100 bp paired-end reads. Raw reads were filtered by trimming adapters and removing low-quality bases with a Q-value of <20 using Cutadapt (Version 1.2.1). Genome mapping was performed using the sequenced strain, *Fructilactobacillus sanfranciscensis* TMW 1.1304. Gene expression levels were calculated using HTSeq (version 0.6.1) and quantified using RPKM. Transcripts with *p* < 0.05 and fold change > 2, as determined by DESeq (version 1.18.0), were considered to be significantly differentially expressed.

### 2.8. Statistical Measurements

All analyses were repeated thrice. The sourdoughs fermented with different *F. sanfranciscensis* strains were compared via the principal component analysis, on the basis of their volatile profiles using XLSTAT software (version 2021.2). IBM SPSS Statistics version 26 was utilized for one-way ANOVA.

## 3. Results and Discussion

### 3.1. Proteolytic Activity of F. sanfranciscensis

**Figure 1:**
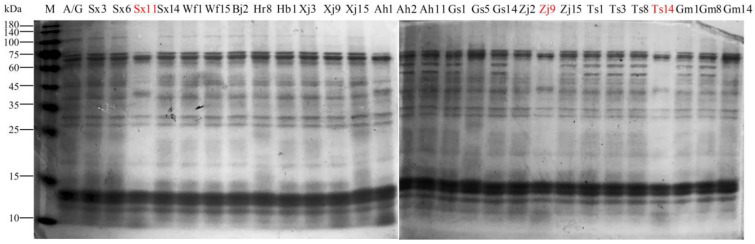
SDS-PAGE analysis of wheat albumins and globulins hydrolyzed by *Fructilactobacillus sanfranciscensis* strains. M, molecular weight standard proteins; A/G, non-hydrolyzed proteins.

Proteolysis during sourdough fermentation changes dough rheology and texture and enhances flavor [29]. Moreover, some gliadin peptides associated with human gluten intolerance can be degraded by select lactobacilli [18]. The proteolytic system in LAB is mainly made up of cell-envelope-associated serine proteinase (CEP), peptide transport systems (Opp, DtpT, and Dpp), and a variety of intracellular peptidases [30,31]. Here, we characterized the proteolytic activities of *F. sanfranciscensis*, including proteinases and peptidases. The proteinase activities of different *F. sanfranciscensis* strains were assayed for wheat albumins and globulins by SDS-PAGE. As shown in Figure 1, most of the strains had no detectable proteinase activity, except for strains Sx11, Zj9, and Ts14, which exhibited significant hydrolysis of albumins and globulins, indicating that *F. sanfranciscensis* strain’s proteinase activity is strain-dependent. These findings are consistent with the research of De Angelis et al. [14]. Furthermore, *F. sanfranciscensis* DSM 20451^T^ and most other sourdough lactobacilli do not have proteinase activity either, according to the results of Vermeulen et al. [32] and Pepe et al. [33]. Nevertheless, endogenous flour proteinases can compensate for or work in concert with lactobacilli to hydrolyze proteinaceous substrates to peptides [18,34].

**Figure 2:**
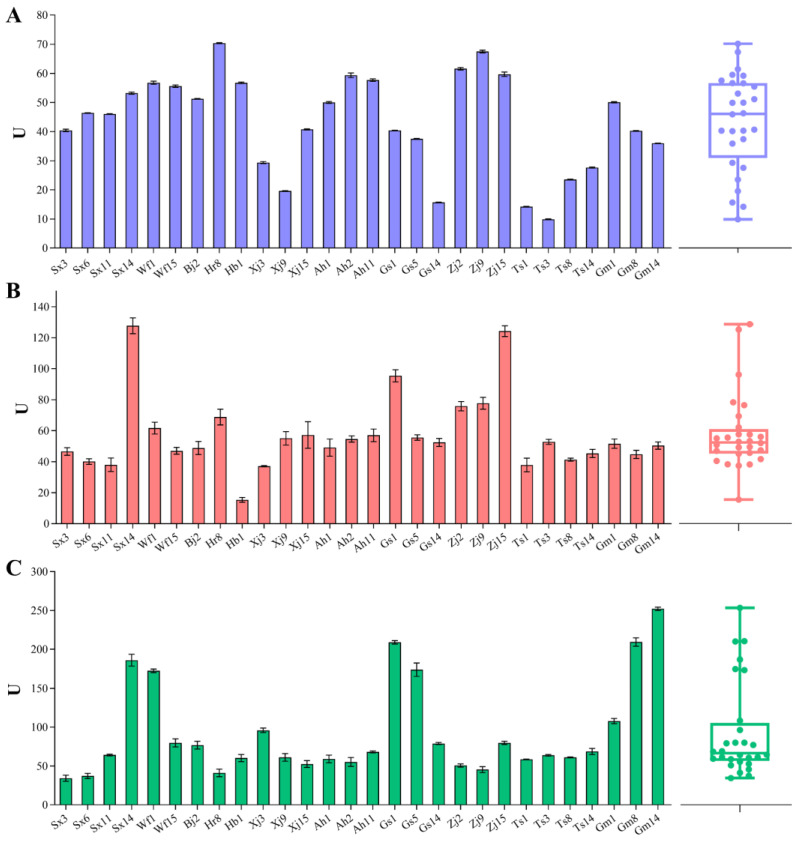
PepN (**A**), PepV (**B**), and PepT (**C**) activities of different *F. sanfranciscensis* strains on Leu-*p*-NA, Leu-Leu, and Leu-Leu-Leu substrates, respectively. Box plots show median values (–), 75th and 25th percentiles of the data (top and bottom of plots, respectively), and maximum and minimum values (top and bottom error bars, respectively). All data points are presented in the plots.

In general, LAB strains have multiple auxotrophs [35]. *F. sanfranciscensis* transports peptides and further degrades them into amino acids, relying on various intracellular peptidases for growth during sourdough fermentation and contributing either directly or indirectly to sourdough characteristics [18]. *F. sanfranciscensis* strains possess the highest aminopeptidase, dipeptidase, tripeptidase, and iminopeptidase activities [17]. We also characterized the peptidase activities of different *F. sanfranciscensis* strains, including PepN (aminopeptidase), PepV (dipeptidase), and PepT (tripeptidase) (Figure 2). All strains exhibited PepN, PepV, and PepT activities. PepN, a general aminopeptidase with broad specificity, has been characterized in diverse LAB strains [36]. This enzyme can hydrolyze the N-terminal amino acids from a wide range of peptides of varying lengths and N-terminal amino acid residues [31]. As shown in Figure 2A, the PepN activity of *F. sanfranciscensis* strains exhibited a wide range from 9.90 ± 0.13 U (Ts3) to 70.36 ± 0.16 U (Hr8). Hr8 and Zj9 were the strains showing the highest activity (70.36 ± 0.16 U and 67.50 ± 0.47 U, respectively). PepV and PepT specifically hydrolyze di- and tripeptides, respectively. These enzymes exhibit broad specificity and prefer di-/tripeptides that contain hydrophobic amino acids such as leucine, phenylalanine, methionine, or glycine [36]. The strains’ PepV activity varied from 15.41 ± 1.60 U (Hb1) to 127.71 ± 5.10 U (Sx14), with 57.51 U as the median value (Figure 2B). Sx14, Zj15, and Gs1 were the strains which possessed the highest PepV activity (127.71 ± 5.10 U, 124.27 ± 3.46 U, and 95.44 ± 3.91 U, respectively). For PepT, the majority of strains showed activities ranging from 40 U to 80 U (Figure 2C). The strains Sx14, Wf1, Gs1, Gs5, Gm8, and Gm14 showed significantly higher activities than the other strains, with Gm14 possessing the highest PepT activity (252.17 ± 2.19 U). These three intracellular peptidases from *F. sanfranciscensis* were characterized in previous research. The genes encoding PepN and PepT were identified in *F. sanfranciscensis* DSM 20451^T^ [32], and PepN and PepV were purified in *F. sanfranciscensis* CB1 [37]. The results in this study illustrated variable peptidase activities among the different *F. sanfranciscensis* strains isolated from traditional sourdoughs, in line with the results of De Angelis et al. [14].

### 3.2. EPS Production by F. sanfranciscensis

**Figure 3:**
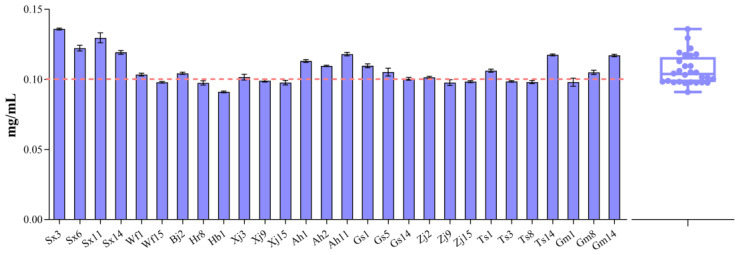
Exopolysaccharide content of *F. sanfranciscensis* strains. Box plots show median values (–), 75th and 25th percentiles of the data (top and bottom of plots, respectively), and maximum and minimum values (top and bottom error bars, respectively). All data points are presented in the plots.

EPSs produced by LAB during sourdough fermentation improve dough rheology and the textural quality of bread and steamed bread [18,38,39]. In addition, Seitter et al. [40] demonstrated that the crude EPS extracts of *F. sanfranciscensis* strains increase stickiness and resistance, but decrease the extensibility of wheat dough. Here, we characterized EPS production in different *F. sanfranciscensis* strains. As shown in Figure 3, most strains produced approximately 0.1 mg/mL of EPS. Sx3 and Sx11 exhibited slightly higher EPS production abilities than the other strains (0.136 ± 0.0007 and 0.130 ± 0.004 mg/mL, respectively). The results indicated no significant difference in EPS production among *F. sanfranciscensis* strains. EPSs are formed by the metabolism of sucrose through the action of a fructosyltransferase enzyme, most likely levansucrase (LevS) in *F. sanfranciscensis* [41,42]. Thus, the slight difference in levansucrase ability or the relevant gene (*levS*) expression in *F. sanfranciscensis* strains may explain their similar EPS production abilities; however, this should be verified in further studies.

### 3.3. Acidification of F. sanfranciscensis during Sourdough Fermentation

**Figure 4:**
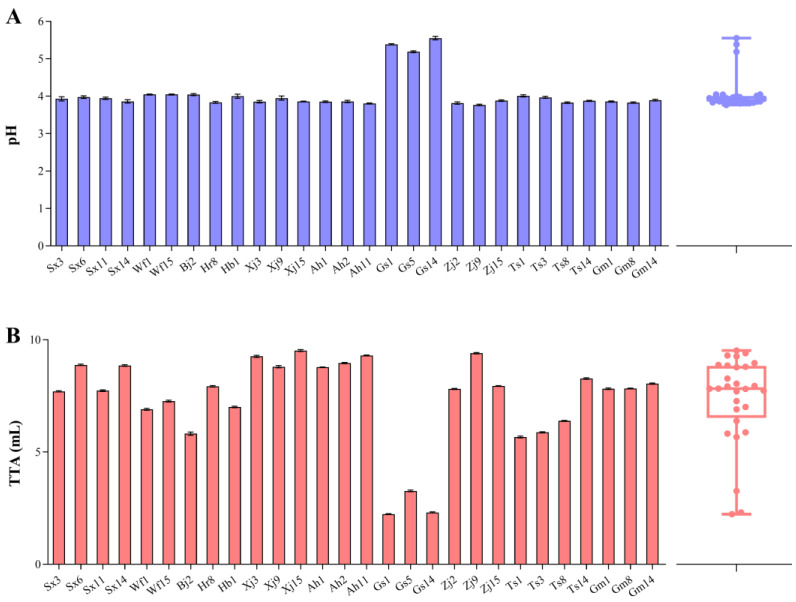
pH (**A**) and TTA (**B**) values of sourdough fermented with *F. sanfranciscensis*. Box plots show median values (–), 75th and 25th percentiles of the data (top and bottom of plots, respectively), and maximum and minimum values (top and bottom error bars, respectively). All data points are presented in the plots.

The pH and TTA of the sourdoughs fermented with different *F. sanfranciscensis* strains were determined (Figure 4). With a few exceptions (Gs1, Gs5, and Gs14), the pH values of sourdough ranged from 3.8 to 4.0. This result was consistent with a previous study in which the pH value of the sourdough fermented with *F. sanfranciscensis* after 12 h was about 4 [43]. Acidity is also related to the degradation of proteins in wheat sourdough as well as the proteolytic activity of sourdough lactobacilli [18]. The low pH could increase the activities of endogenous wheat proteinases, which are optimum at pH 3.0–4.0 and enhance the proteolytic breakdown of cereal proteins [44,45]. The TTA values of the sourdoughs varied from 2.23 ± 0.02 (Gs1) to 9.52 ± 0.04 (Xj15), indicating differences in the acidification capacity of *F. sanfranciscensis* strains, which agrees with the results of previous research [14]. Xj15, Zj9, Ah11, and Xj3 were the strains with the highest acidifying activities (9.52 ± 0.04, 9.40 ± 0.03, 9.30 ± 0.02, and 9.26 ± 0.05, respectively). Strains Gs1, Gs5, and Gs14 showed the lowest acidification capabilities (2.23 ± 0.02, 3.27 ± 0.04, and 2.31 ± 0.03, respectively), in line with the pH results. Notably, some sourdoughs with similar pH values differed significantly in their TTA values, such as Xj15 and Ts8, and Sx6 and Ts3. The various types of organic acids produced by different *F. sanfranciscensis* strains may explain this observation; however, further investigation is required.

**Figure 5:**
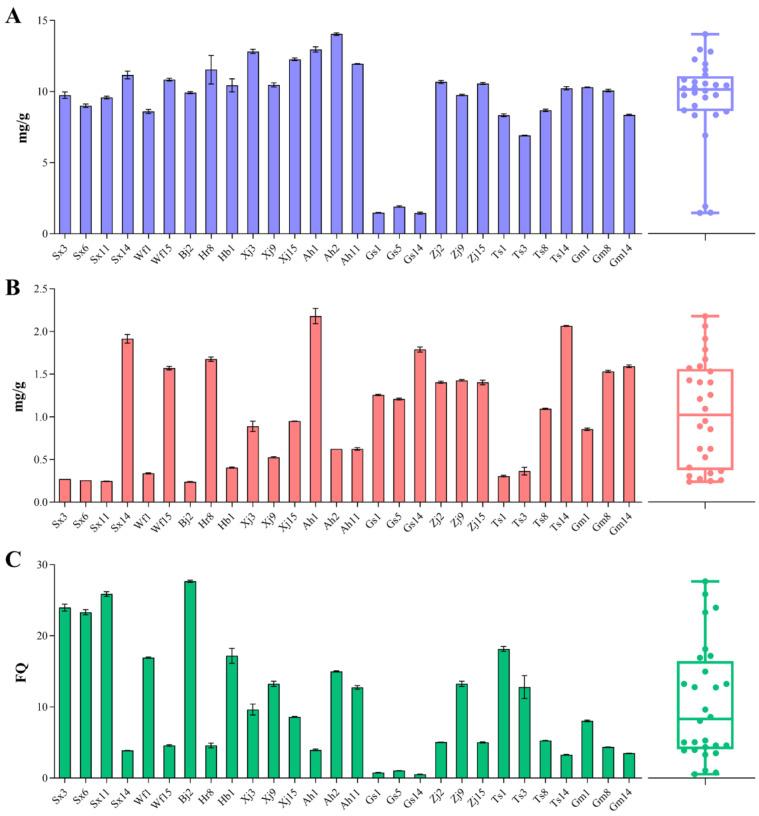
Concentrations of lactic (**A**) and acetic (**B**) acids and fermentation quotient (**C**) of sourdoughs fermented by *F. sanfranciscensis* strains. Box plots show median values (–), 75th and 25th percentiles of the data (top and bottom of plots, respectively), and maximum and minimum values (top and bottom error bars, respectively). All data points are presented in the plots.

Lactic and acetic acids, two major acids produced by LAB, are important for the sensory qualities of sourdoughs [18]. Therefore, we determined lactic and acetic acid levels in each sourdough sample. As shown in Figure 5, lactic and acetic acid synthesis varied among the different *F. sanfranciscensis* strains. The concentration of lactic acid ranged from 1.45 ± 0.07 (Gs14) to 14.04 ± 0.09 (Ah2) mg/g. Strains Ah2, Ah1, and Xj3 showed the highest abilities to produce lactic acid (14.04 ± 0.09, 12.96 ± 0.19, and 12.82 ± 0.15 mg/g, respectively). In addition, the sourdoughs fermented by Gs1, Gs5, and Gs14 exhibited the lowest lactic acid concentrations, indicating that a weak ability to produce lactic acid might be the main reason for the significant difference in the pH and TTA values of their fermented sourdoughs compared to those of other strains. Acetic acid is regarded as a crucial flavor enhancer that, along with lactic acid, catalyzes the Maillard reaction [46]. The concentration of acetic acid varied substantially from 0.24 ± 0.003 (Bj2) to 2.18 ± 0.090 (Ah1) mg/g, with a median value of 1.04 mg/g. Ah1, Ts14, and Sx14 were the strains showing the highest abilities to produce acetic acid (2.18 ± 0.090, 2.06 ± 0.006, and 1.92 ± 0.05 mg/g, respectively). The lactic-to-acetic acid molar ratio, defined as the fermentation quotient (FQ), is a fermentative parameter widely used to link acidity and flavor [14,18]. The most recommended value of FQ is below 5.0 for sourdough fermentation [2]. Variations in lactic and acetic acid contents determined the differences in FQ. As shown in Figure 5C, FQ exhibited large fluctuations from 0.54 ± 0.02 (Gs14) to 27.67 ± 0.15 (Bj2), with a median value of 10.14. Similar results can be found in the previous study performed by De Angelis et al. [14]. Strains Bj2, Sx11, Sx3, and Sx6 showed the highest FQ (27.67 ± 0.15, 25.88 ± 0.34, 23.97 ± 0.49, and 23.33 ± 0.35, respectively), whereas Gs14, Gs1, and Gs5 had the lowest value (0.54 ± 0.02, 0.78 ± 0.01, and 1.06 ± 0.01, respectively). Among all strains, 46.4% had FQ < 5. In general, a combination of obligate and facultative heterofermentative LAB species is required to obtain an ideal FQ [47]. However, according to the results of this study, some *F. sanfranciscensis* strains alone could appear to be good candidates for optimal FQ.

### 3.4. Volatile Compound Profiles

**Figure 6:**
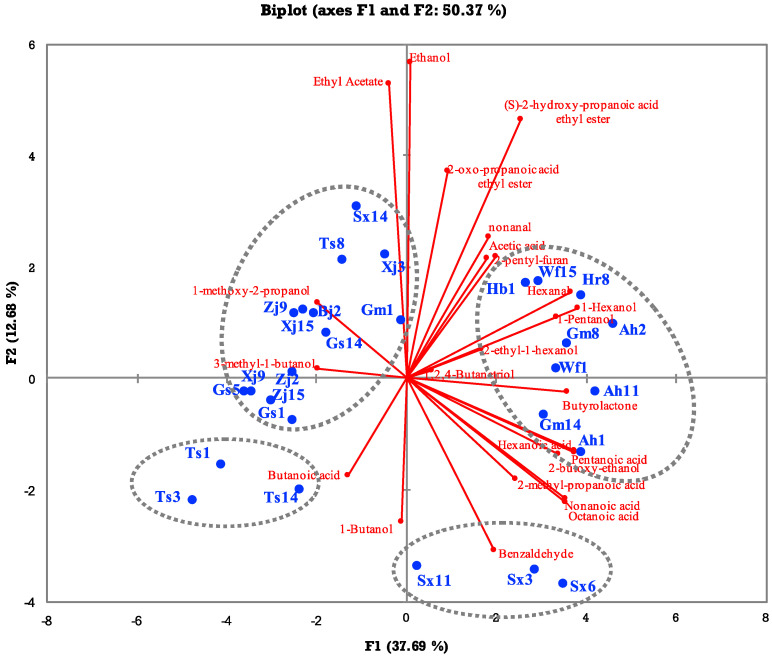
Principal component analysis biplot showing volatile compounds in sourdoughs fermented with 28 *F. sanfranciscensis* strains. Samples divided into the same group are marked by circles.

The volatile profiles of *F. sanfranciscensis* strains are broad and uniform, differing greatly from those of other heterolactic species [18,43]. In this study, twenty-four volatile compounds were determined in sourdoughs fermented with twenty-eight *F. sanfranciscensis* strains, including nine alcohols, seven acids, four esters, three aldehydes, and one furan. Compounds with a score <90 compared to the NIST 17 database were removed. Ethyl acetate, alcohols (ethanol, 1-butanol, 1-methoxy-2-propanol, 3-methyl-1-butanol, 1-pentanol, and 1-hexanol), aldehydes (hexanal and nonanal), and acids (acetic acid, butanoic acid, pentanoic acid, hexanoic acid, and nonanoic acid) were the main volatile compounds in the sourdoughs fermented with *F. sanfranciscensis* strains, in accordance with the results previously reported by Liu et al. [43]. Principal component analysis (PCA) was carried out to provide an overview of differences in the flavor of sourdough samples (Figure 6). The first two principal components explained 37.69% and 12.68% of the variation. As Figure 6 depicts, the samples were categorized into four groups. Samples fermented with the strains Sx14, Ts8, Gm1, Xj3, Zj9, Xj15, Bj2, Gs14, Xj9, Zj2, Gs5, Zj15, and Gs1 were grouped together and featured relatively high contents of ethanol, 1-methoxy-2-propanol, and ethyl acetate. Ethyl acetate, one of the main compounds produced by *F. sanfranciscensis* [17], is positively correlated with the aroma of sourdough and steamed bread because it possesses pleasant, sweet, and fruity odors [48]. Ethanol is reported to be the most abundant aromatic compound in many sourdoughs [49,50]. According to Hansen and Hansen [51], sourdoughs fermented by *F. sanfranciscensis* produced the highest quantities of ethanol and ethyl acetate. Wu et al. [4] also supported the abundance of ethanol when investigating the diversity of volatiles in the sourdough fermented with *L. plantarum*, *L. brevis*, and *F. sanfranciscensis*. Samples fermented with the strains Hb1, Wf15, Hr8, Ah2, Gm8, Wf1, Ah11, Gm14, and Ah1 were grouped together and featured relatively high amounts of nonanal, acetic acid, 2-pentyl-furan, hexanal, pentanol, pentanoic acid, and 2-methyl-propanoic acid. Nonanal and 2-pentyl-furan contribute to pleasant aromas in wheat bread, whereas hexanal and 2-methyl-propanoic acid contribute to unpleasant aromas [48,52]. Except for the three acids, these compounds reportedly originate from the oxidation of flour lipids [53]. Nevertheless, their content in sourdough could be affected by LAB. Some aldehydes have been reported to be utilized as electron acceptors and reduced by LAB in sourdough fermentation [54]. The different abilities of *F. sanfranciscensis* strains to reduce aldehydes may lead to the variety in the concentration of these compounds among sourdough samples. The samples fermented with Sx11, Sx3, and Sx6 possessed relatively high concentrations of benzaldehyde, whereas those fermented with Ts1, Ts3, and Ts14 contained relatively high concentrations of butanoic acid. Benzaldehyde, derived from lipid oxidation or fermentation by certain LAB [53], is characterized by an almond and caramel-like odor [48]. Butanoic acid, the most abundant in sourdough fermented with Ts14, contributes to an unpleasant aroma in bread, characterized by a sweaty and rancid odor [48,55].

According to the results, some strains isolated from the same sourdough were clustered together and shared a similar volatile profile even though they belonged to different genotypic clusters, such as Xj3, Xj9, and Xj15; Gs1, Gs5, and Gs14; Ah1, Ah2, and Ah11; and Sx3, Sx6, and Sx11, indicating that the volatile profiles of *F. sanfranciscensis* strains seemed to have some relation to their regional origin. Nevertheless, the strains Sx14, Ts8, and Gm1 were separate from the other isolates of the same sourdough on the principal component analysis biplot of volatiles. Moreover, strains isolated from sourdoughs in different regions were grouped together based on their aroma profiles, like Sx14, Ts8, Gm1, Xj3, Zj9, Gs1, and Bj2, and Hb1, Wf15, Hr8, Ah2, and Gm8. Thus, more different strains isolated from sourdoughs in different regions should be sampled and analyzed to gain a deep insight into the correlation between the volatile profile and geographical origin of *F. sanfranciscensis* strains in further studies.

### 3.5. Gene Expression Analysis of Different F. sanfranciscensis Strains during Sourdough Fermentation

Based on our phenotypic trait analysis of *F. sanfranciscensis*, the strains Sx14 and Ts1 showed significant differences in peptidase activities and the amount of acidification and volatiles in their fermented sourdoughs. Moreover, they were isolated from sourdoughs in different regions and also belonged to diverse genetic clusters. Therefore, these two strains were chosen for mRNA sequencing to gain a deep insight into their intraspecies diversity during sourdough fermentation. Transcriptome differentiation between *F. sanfranciscensis* Sx14 and Ts1 after 12 h fermentation in sourdough was determined. The data indicated that the expression of 524 genes significantly differed between the two strains, with 261 upregulated and 263 downregulated genes (Sx14 vs. Ts1). Table 2 illustrates the lists of differentially expressed genes with at least a three-fold change between strains Sx14 and Ts1. GO and KEGG enrichment analysis of the differential genes was also performed. The significantly enriched GO terms and KEGG pathways are shown in Table 3 and Table 4, respectively. The *malL* and *glf* genes, encoding oligo-1,6-glucosidase and UDP-galactopyranose mutase, respectively, both of which are associated with the metabolism of galactose, were the most upregulated genes. Galactose metabolism was a significantly enriched pathway, as shown by KEGG analysis (Table 4), and the upregulations of these two genes in strain Sx14 may suggest enhanced galactose utilization by Sx14 when compared to Ts1. The *pepDA* and *map* genes, encoding dipeptidase and type I methionyl aminopeptidase, respectively, were both significantly upregulated in strain Sx14, indicating higher activities of these two peptidases in Sx14 than in Ts1. In addition, the genes encoding L-lactate dehydrogenase (*ldh*) and acetate kinase (*ackA*), two key enzymes in the synthesis of lactic and acetic acids, respectively [56], showed higher mRNA levels in Sx14 than in Ts1. These results indicate that *F. sanfranciscensis* Sx14 produced more lactic and acetic acids during sourdough fermentation than strain Ts1, which agrees with the acidification analysis of *F. sanfranciscensis*.

In contrast, some gene’s transcript levels were markedly reduced in the strain Sx14 relative to Ts1. The genes *rpsL*, *rpsG*, *rplC*, *rplK*, *rplL*, *rplV*, *rplB*, and *rplN*, which encode diverse ribosomal subunit proteins, were significantly upregulated in Ts1. As shown in Table 3, GO terms related to the activities of ribosome were significantly enriched. Consistent with the GO analysis, the most enriched KEGG pathway was ribosome. The upregulations of these genes may indicate higher growth and proliferation abilities in Ts1 after 12 h fermentation [57]. However, it should be verified by growth experiments of these two strains. ATP-binding cassette (ABC) transporters were also a significantly enriched KEGG pathway, as shown in Table 4. Genes involved in ABC transporters and mediating ATP-powered transmembrane transport of substrates, including *glnM*, *glnH*, *metQ*, and *ecfA1*, were significantly upregulated in Ts1. Among them, *glnM* and *glnH* encode aspartate/glutamate/glutamine transport system permease and substrate-binding proteins, respectively, whereas *metQ* encodes a D-methionine transport system substrate-binding protein. This may indicate greater transmembrane transport of substrates, especially aspartate, glutamate, glutamine, and D-methionine, in Ts1 than in Sx14. Furthermore, *scrK*, which encodes fructokinase and is involved in fructose metabolism [56], exhibited a higher transcript level in Ts1 than in Sx14, suggesting enhanced utilization of fructose as a carbon source by Ts1.

## 4. Conclusions

In this study, we revealed differences in the proteinase and peptidase abilities of *F. sanfranciscensis* strains, and the acidification and volatiles of sourdough fermented with these strains. Several strains were characterized by marked proteinase (e.g., Sx11, Zj9, and Ts14) and/or peptidase activities (e.g., Hr8, Sx14, and Gm14), and some strains alone appeared to be good candidates for optimal FQ. The gene expression of different *F. sanfranciscensis* strains during sourdough fermentation indicated that the strain Sx14 possessed higher dipeptidase and aminopeptidase activities, as well as higher galactose utilization and lactic and acetic acid production abilities, whereas Ts1 showed higher transmembrane transport of substrates and fructose utilization. Moreover, it seems that higher growth and proliferation abilities were shown in Ts1 than in Sx14 after 12 h fermentation through transcriptome analysis, but this should be confirmed by growth experiments. In further studies, the transcriptome of additional strains from different sourdoughs and regions as well as different time points during sourdough fermentation will be throughly investigated to better reveal the intraspecies diversity of *F. sanfranciscensis*.

## Figures and Tables

**Table 1:** Information of *F. sanfranciscensis* strains employed in this study according to Yang et al. [16].

Organism	Strains	Isolation Source	Genetic Clusters *
*F* *. sanfranciscensis*	Wf1	Sourdough, Shandong China	1
*F* *. sanfranciscensis*	Wf15	Sourdough, Shandong China	1
*F* *. sanfranciscensis*	Gm1	Sourdough, Shandong China	2
*F* *. sanfranciscensis*	Gm8	Sourdough, Shandong China	3
*F* *. sanfranciscensis*	Gm14	Sourdough, Shandong China	4
*F* *. sanfranciscensis*	Ah1	Sourdough, Anhui China	4
*F* *. sanfranciscensis*	Ah2	Sourdough, Anhui China	3
*F* *. sanfranciscensis*	Ah11	Sourdough, Anhui China	5
*F* *. sanfranciscensis*	Sx3	Sourdough, Shanxi China	6
*F* *. sanfranciscensis*	Sx6	Sourdough, Shanxi China	7
*F* *. sanfranciscensis*	Sx11	Sourdough, Shanxi China	8
*F* *. sanfranciscensis*	Sx14	Sourdough, Shanxi China	9
*F* *. sanfranciscensis*	Gs1	Sourdough, Gansu China	10
*F* *. sanfranciscensis*	Gs5	Sourdough, Gansu China	11
*F* *. sanfranciscensis*	Gs14	Sourdough, Gansu China	12
*F* *. sanfranciscensis*	Bj2	Sourdough, Shannxi China	1
*F* *. sanfranciscensis*	Zj2	Sourdough, Zhejiang China	13
*F* *. sanfranciscensis*	Zj9	Sourdough, Zhejiang China	13
*F* *. sanfranciscensis*	Zj15	Sourdough, Zhejiang China	14
*F* *. sanfranciscensis*	Hb1	Sourdough, Hebei China	1
*F* *. sanfranciscensis*	Ts1	Sourdough, Hebei China	15
*F* *. sanfranciscensis*	Ts3	Sourdough, Hebei China	16
*F* *. sanfranciscensis*	Ts8	Sourdough, Hebei China	17
*F* *. sanfranciscensis*	Ts14	Sourdough, Hebei China	16
*F* *. sanfranciscensis*	Hr8	Sourdough, Heilongjiang China	14
*F* *. sanfranciscensis*	Xj3	Sourdough, Xinjiang China	18
*F* *. sanfranciscensis*	Xj9	Sourdough, Xinjiang China	19
*F* *. sanfranciscensis*	Xj15	Sourdough, Xinjiang China	20

* The strains with the same genotypes revealed by both multiplex-RAPD and MLST were grouped into one genetic cluster.

**Table 2:** The most differentially expressed genes in *F. sanfranciscensis* Sx14 and Ts1 during sourdough fermentation (Sx14 vs. Ts1).

Gene_ID	Description	Fold Change	*p*-Value
Upregulated			
*malL*	oligo-1,6-glucosidase	207.63	<0.001
*glf*	UDP-galactopyranose mutase	72.07	<0.001
*gntK*	gluconokinase	13.59	<0.001
*gpo*	glutathione peroxidase	12.92	<0.001
*clpE*	ATP-dependent Clp protease ATP-binding subunit	8.30	<0.001
*LSA_RS04355*	class I SAM-dependent methyltransferase	8.04	<0.001
*pepDA*	dipeptidase	7.55	<0.001
*dnaI*	primosomal protein DnaI	7.41	<0.001
*nrdI*	ribonucleotide reductase assembly protein NrdI	7.16	<0.001
*plsC*	1-acyl-sn-glycerol-3-phosphate acyltransferase	6.51	<0.001
*ysgA*	RNA methyltransferase	6.13	<0.001
*ecsA*	ABC transporter ATP-binding protein	5.96	<0.001
*thyA*	thymidylate synthase	5.77	<0.001
*gor*	glutathione reductase	5.26	<0.001
*npdA*	NAD-dependent protein deacylase	4.98	<0.001
*guaA*	glutamine-hydrolyzing GMP synthase	4.63	<0.001
*ackA*	acetate kinase	4.30	<0.001
*pyrD*	dihydroorotate dehydrogenase	3.93	<0.001
*rluD*	RluA family pseudouridine synthase	3.75	<0.001
*citD*	citrate lyase subunit gamma (acyl carrier protein)	3.74	<0.001
*ybeY*	rRNA maturation RNase YbeY	3.61	<0.001
*LSA_RS04400*	DUF948 domain-containing protein	3.17	<0.001
*ldh*	L-lactate dehydrogenase	3.08	<0.001
*gntR*	GntR family transcriptional regulator	3.04	<0.001
*map*	type I methionyl aminopeptidase	3.01	<0.001
Downregulated			
*glnM*	aspartate/glutamate/glutamine transport system permease protein	23.65	<0.001
*glnH*	aspartate/glutamate/glutamine transport system substrate-binding protein	18.84	<0.001
*rpsL*	small subunit ribosomal protein S12	13.42	<0.001
*rpsG*	small subunit ribosomal protein S7	9.23	<0.001
*ltaS*	LTA synthase family protein	8.90	<0.001
*typA*	translational GTPase TypA	8.82	<0.001
*metQ*	D-methionine transport system substrate-binding protein	8.12	<0.001
*LSA_RS06240*	membrane protein	7.63	<0.001
*accD*	acetyl-CoA carboxylase subunit beta	7.11	<0.001
*fusA*	elongation factor G	6.57	<0.001
*lytR*	LytR family transcriptional regulator	6.04	<0.001
*rplC*	50S ribosomal protein L3	5.63	<0.001
*rplK*	large subunit ribosomal protein L11	5.08	<0.001
*minD*	septum site-determining protein MinD	4.90	<0.001
*LSA_RS01620*	threonine/serine exporter	4.63	0.005
*rplL*	large subunit ribosomal protein L7/L12	4.41	<0.001
*LSA_RS01505*	cation:proton antiporter	4.25	0.008
*rplV*	50S ribosomal protein L22	4.05	<0.001
*ecfA1*	energy-coupling factor transport system ATP-binding protein	3.94	<0.001
*adk*	adenylate kinase	3.64	<0.001
*rplB*	large subunit ribosomal protein L2	3.47	<0.001
*prsA*	peptidylprolyl isomerase	3.33	0.015
*rplN*	large subunit ribosomal protein L14	3.13	<0.001
*scrK*	fructokinase	3.11	<0.001
*LSA_RS05375*	gfo/Idh/MocA family oxidoreductase	3.08	<0.001
*adh2*	bifunctional acetaldehyde-CoA/alcohol dehydrogenase	3.02	<0.001

**Table 3:** The functional classification of the significantly differentially expressed genes in *F. sanfranciscensis* Sx14 and Ts1 by GO enrichment.

Catergory	GO_Term	Cluster Frequency	Corrected *p*-Value
molecular_function	GO:0019843 rRNA binding	7.0%	<0.001
cellular_component	GO:0005840 ribosome	8.5%	0.003
cellular_component	GO:0030529 intracellular ribonucleoprotein complex	8.5%	0.005
cellular_component	GO:1990904 ribonucleoprotein complex	8.5%	0.006
cellular_component	GO:0044391 ribosomal subunit	2.7%	0.01
molecular_function	GO:0005198 structural molecule activity	8.0%	0.03

**Table 4:** KEGG enrichment of the differentially expressed genes in *F. sanfranciscensis* Sx14 and Ts1.

Pathway	*p*-Value
Ribosome	<0.001
Starch and sucrose metabolism	0.014
Galactose metabolism	0.021
Two-component system	0.028
ABC transporters	0.035
Arginine biosynthesis	0.041

## Data Availability

The original contributions presented in the study are included in the article, further inquiries can be directed to the corresponding author.
